# Detecting Asymptomatic Infections of Rice Bacterial Leaf Blight Using Hyperspectral Imaging and 3-Dimensional Convolutional Neural Network With Spectral Dilated Convolution

**DOI:** 10.3389/fpls.2022.963170

**Published:** 2022-07-13

**Authors:** Yifei Cao, Peisen Yuan, Huanliang Xu, José Fernán Martínez-Ortega, Jiarui Feng, Zhaoyu Zhai

**Affiliations:** ^1^College of Artificial Intelligence, Nanjing Agricultural University, Nanjing, China; ^2^College of Engineering, Nanjing Agricultural University, Nanjing, China; ^3^Departamento de Ingeniería Telemática y Electrónica (DTE), Escuela Técnica Superior de Ingeniería y Sistemas de Telecomunicación, Universidad Politécnica de Madrid (UPM), Madrid, Spain

**Keywords:** bacterial leaf blight, asymptomatic infection, hyperspectral imaging, deep learning, spectral dilated convolution, interpretable

## Abstract

Rice is one of the most important food crops for human beings. Its total production ranks third in the grain crop output. Bacterial Leaf Blight (BLB), as one of the three major diseases of rice, occurs every year, posing a huge threat to rice production and safety. There is an asymptomatic period between the infection and the onset periods, and BLB will spread rapidly and widely under suitable conditions. Therefore, accurate detection of early asymptomatic BLB is very necessary. The purpose of this study was to test the feasibility of detecting early asymptomatic infection of the rice BLB disease based on hyperspectral imaging and Spectral Dilated Convolution 3-Dimensional Convolutional Neural Network (SDC-3DCNN). First, hyperspectral images were obtained from rice leaves infected with the BLB disease at the tillering stage. The spectrum was smoothed by the Savitzky–Golay (SG) method, and the wavelength between 450 and 950 nm was intercepted for analysis. Then Principal Component Analysis (PCA) and Random Forest (RF) were used to extract the feature information from the original spectra as inputs. The overall performance of the SDC-3DCNN model with different numbers of input features and different spectral dilated ratios was evaluated. Lastly, the saliency map visualization was used to explain the sensitivity of individual wavelengths. The results showed that the performance of the SDC-3DCNN model reached an accuracy of 95.4427% when the number of inputs is 50 characteristic wavelengths (extracted by RF) and the dilated ratio is set at 5. The saliency-sensitive wavelengths were identified in the range from 530 to 570 nm, which overlaps with the important wavelengths extracted by RF. According to our findings, combining hyperspectral imaging and deep learning can be a reliable approach for identifying early asymptomatic infection of the rice BLB disease, providing sufficient support for early warning and rice disease prevention.

## Introduction

Rice is one of the most important grain crops, more than half of the world’s population relies on it for food (Wu et al., 2020). Achieving steady and high rice yield has always been the goal of agricultural production. Bacterial leaf blight (BLB) disease, as one of the three major diseases of rice, is not evenly distributed in rice fields, but occurs in patches with large areas of the field free of disease in the early stages of infestation. In recent years, the outbreak area of BLB disease accounts for about one third of the total planting areas, and the average diseased plant rate is about 10%. It has a huge impact on the yield of rice. Generally, the yield is reduced by up to 50–60%, and even the grains are not harvested (Zarco-Tejada et al., 2018; Zhang et al., 2020). To reduce the negative effects of rice BLB disease, farmers used to treat a large number of pesticides. Overuse of pesticides not only increases the treatment expenditure, but also pollutes the environment (Šebela et al., 2018; Zhang J. et al., 2019). From the perspective of plant protection, the primary task is to quickly and accurately identify the potential occurrence of BLB, and then apply chemical treatments with the required amount (Tian et al., 2021). Therefore, the early identification of BLB is particularly critical. Early detection and prevention, as well as timely guidance, enable farmers to take efficient measures to control the spread of the disease, thereby reducing the amount of pesticides applied, and achieving the goal of sustainable agriculture (Rahman et al., 2020; Shu et al., 2021).

As computer vision and deep learning techniques have developed rapidly in recent years, they have shown great promise in detecting plant diseases (Qiu et al., 2021). Various rice disease detection methods have been proposed by detecting external changes of infected rice leaves from RGB images. Jiang et al. (2021) used a VGG-16 model and RGB images to recognize diseases of rice leaves and wheat leaves at the same time. Lu et al. (2017) proposed a deep convolutional neural network to identify a dataset of 500 RGB images containing 10 rice diseases, and the accuracy achieved at 95.48%. Besides, researchers have applied evolutionary approaches to neural architecture search of convolutional neural networks for improving computational efficiency (Xue et al., 2021a,b).

**FIGURE 1:**
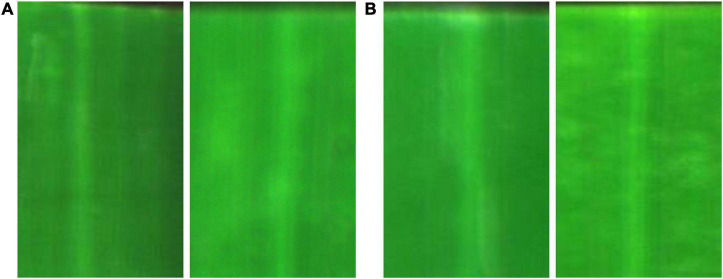
RGB images of rice leaves: **(A)** healthy; **(B)** asymptomatic.

Although deep neural networks have achieved great success in detecting rice disease from RGB images, it is worth noting that these networks may fail to generate correct results for the early asymptomatic BLB disease detection based on RGB images. The examples of healthy and asymptomatic leaves are presented in Figure 1. Without giving labels in advance, one can hardly identify whether the leaf is healthy (Figure 1A) or asymptomatic (Figure 1B) due to their similar visual textures.

**FIGURE 2:**
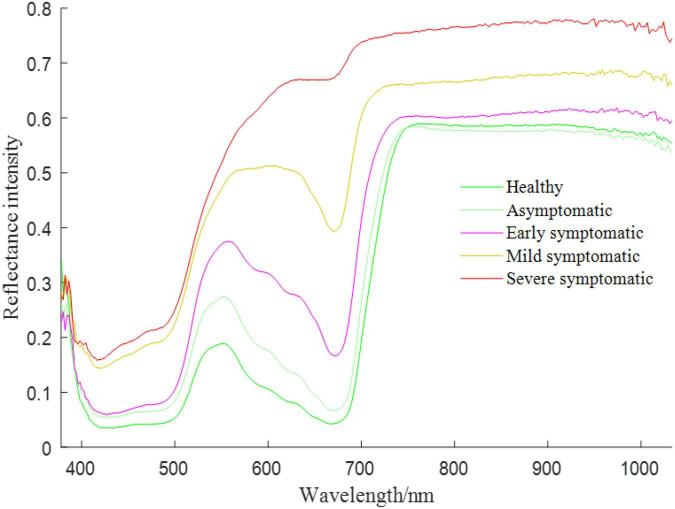
Spectra of infected leaves under different stages.

Since the rice BLB disease is caused by pathogen, rice leaves under such a stress would experience two periods, including asymptomatic and symptomatic stages (Deng et al., 2019; Tian et al., 2021). Although there are no significant lesions shown at the asymptomatic stage, the inner chemical composition has changed according to the plant pathology theory (Chen et al., 2020). Therefore, this inner change in rice leaves motivated us to adopt the hyperspectral imaging technique to detect the asymptomatic infection of the rice BLB disease and we obtained the following results shown in Figure 2. It can be seen that there are distinguishable features between the wavelengths from 378 to 1033 nm of hyperspectral images of healthy and infected rice leaves. This finding motivates us to use the hyperspectral imaging technique to detect the rice BLB disease at the asymptomatic stage, thereby providing an earlier warning to the farmers and assisting them in decision making about chemical treatments.

Hyperspectral imaging is a technique that analyzes multiple wavelengths of reflectance intensity of each pixel instead of just investigating primary colors (e.g., red, green, and blue) that only cover the wavelength range from 450 to 680 nm (Lowe et al., 2017; Zhang et al., 2021a). In comparison to traditional spectral and image technologies, hyperspectral imaging technology can obtain multiple wavelengths of spectrum and image information at the same time, and it has been demonstrated to be an effective and non-destructive technique for detecting crop diseases across multiple scales (Franceschini et al., 2019; Riefolo et al., 2021). Adopting the hyperspectral imaging technique to detect leaf disease has become a popular approach because hyperspectral imaging has high potential for finding new insights about plant diseases. Through the information fusion of multiple wavelengths, hyperspectral imaging can achieve better classification performance than using RGB images (Cabrera Ardila et al., 2020; Wang et al., 2021). Koushik et al. (2019) used hyperspectral imaging to detect soybean charcoal rot disease. Zhao et al. (2020) combined hyperspectral imaging and SVM to detect wheat early powdery mildew. Zhang et al. (2021a) applied in-situ hyperspectral imaging to diagnose the symptoms of sheath blight disease on rice stalk. Kaitlin et al. (2020) detected potato pre-symptomatic of late blight and early blight by hyperspectral imaging. From above works, it can be concluded that hyperspectral imaging is a powerful technique to detect the early disease of plants. Meanwhile, it is also worth noting that the high dimensionality of hyperspectral images might bring a huge challenge to the computational complexity, which is an urgent problem to be solved in the detection of plant diseases using hyperspectral images (Zhang et al., 2020).

Because of the high dimensionality of hyperspectral images, shallow machine learning models cannot perfectly handle the target detection tasks in hyperspectral images (Kaitlin et al., 2020). Deep learning, on the other hand, has shown its great potential in diverse applications. Hyperspectral images can be treated as a stack of 2D matrices, representing the correlations between spatial and spectral directions (Zhang J. et al., 2019). Therefore, many scholars have contributed to the combination of deep learning and hyperspectral imaging for plant disease identification (Polder et al., 2019; Xiao et al., 2022). Chu et al. (2022) proposed a shallow convolutional neural network with attention mechanism model to predict the early herbicide stress in wheat cultivars. Polder et al. (2019) designed a new imaging setup consisting of a hyperspectral line-scan camera and applied a convolutional neural network for detecting potato virus Y. Nguyen et al. (2021) applied both deep learning and machine learning models to identify grapevine early vein-clearing virus in hyperspectral images. The result showed that the former achieved a better classification result. Conclusively, combining deep learning with hyperspectral imaging is very promising in the plant disease detection task.

The purpose of this paper is to apply hyperspectral imaging and deep learning to detect asymptomatic infections of the rice BLB disease. The main contributions of this paper were summarized as follows: (1) to explore the applicability of using random forest (RF) and principal component analysis (PCA) to extract sensitive features from raw hyperspectral data, thereby improving the computational efficiency; (2) to build a spectral dilated convolution 3-dimensional convolutional neural network (SDC-3DCNN) model and test the effect of the number of input features on the detection performance; (3) to assess the detection performance of the SDC-3DCNN model under different spectral dilated ratios (SDR); (4) to interpret the important wavelengths with a saliency map method. In conclusion, the proposed SDC-3DCNN model is able to detect the asymptomatic infection of the rice BLB disease, thereby providing early warnings before the disease outbreak. This result may assist in arranging chemical treatments for disease control.

## Materials and Methods

### Experimental Materials

#### Rice Planting and Artificial Inoculation

The experimental materials were processed and collected in 2020 at a greenhouse base in Nanjing Agricultural University, Nanjing, Jiangsu, China. The rice seeds were sown and then transplanted into plastic pots (three plants per pot) on June 17th, 2020. The rice was grown in plastic pots (35 cm diameter × 32 cm height) filled with paddy clay soil. A total of 50 pots were used for the hyperspectral data collection. Data collected from different rice leaves provided an opportunity to evaluate the reproducibility and reliability of the findings on disease detection. To ensure consistent management practices in the greenhouse environment, the basal nutrition fertilizers (nitrogen provided by urea, 150 kg⋅ha^–2^; P_2_O_5_, 135 kg⋅ha^–2^; and K_2_O, 18.3 kg⋅ha^–2^) were applied prior to transplantation, and a second nitrogen topdressing (N, 150 kg⋅ha^–2^) was applied during the tillering stage. The rice plants were irrigated as needed to ensure that the soil in each pot was always covered by a shallow layer of water. All plants were placed outdoors and were not transferred to the greenhouse until 1 week before the inoculation treatment. The greenhouse comprised two layers of transparent materials and was equipped with air conditioning and humidifying facilities to provide suitable environmental conditions (26–32°C, over 90% relative humidity, and a photoperiod of 14 h) for the artificial inoculation of the BLB pathogen. The artificial inoculation operations were conducted on rice leaves to induce BLB infection at the tillering stage. The BLB pathogen (*Xanthomonas oryzae pv.oryzae Xoo*) was provided by the College of Plant Protection at Nanjing Agricultural University, Nanjing, Jiangsu, China. After the BLB pathogen was isolated, it was transferred to the plate medium (beef extract 0.3%, meat peptone 1%, sucrose 1%, and agar 2%). The bacteria were placed in an incubator at 28°C and cultivated for 48 h, diluted with phosphate buffered saline. The concentration was diluted to about 9 × 10^9^ bacteria per milliliter by the turbidimetric method. The surgical scissors were used to dip the bacterial solution and cut off the tip of the rice flag leaf to complete the bacterial inoculation. All inoculation operations were completed within 3 h. After inoculation, all plants were completely covered with black, light-tight plastic materials for 48 h. Temperature (26–32°C), relative humidity (≥90%), and light conditions were all strictly controlled to ensure a successful infection.

#### Definition of the Disease Infection Process

Previous studies have typically defined disease severity at the leaf level as the average percentage of symptomatic surface areas (Bock et al., 2020; Tian et al., 2021). However, rice leaves with asymptomatic infections cannot be described by this method. For the rice BLB disease, the appearance of obvious symptoms on rice leaves indicates that a large area has begun to spread, which is not conducive to the timely prevention of the disease. Effectively identifying asymptomatic infections is crucial for the prevention and control of the BLB disease.

**FIGURE 3:**
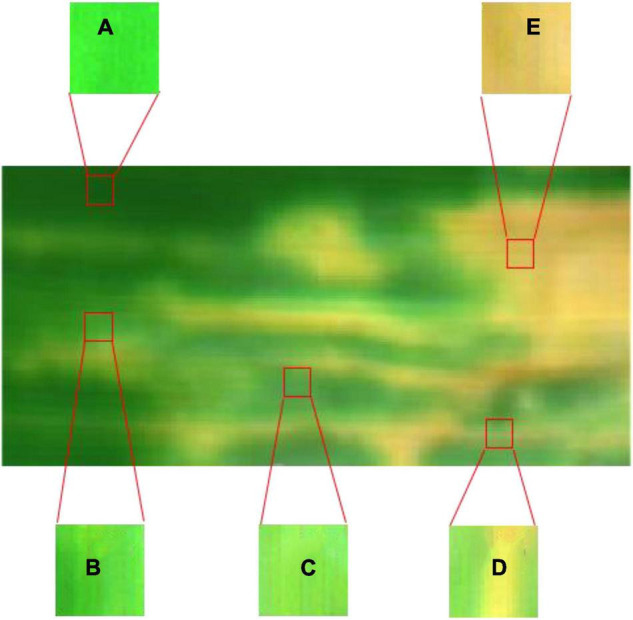
The pixel-level annotation of disease severity levels. **(A)** Denotes pixels that belong to the healthy class. **(B)** Denotes pixels that belong to the asymptomatic class. **(C–E)** Denote pixels that belong to the symptomatic class.

Since the spectrum of health pixels is different from infected areas, a pixel-level annotation method can be used to visualize areas of asymptomatic infection and to more precisely define the disease levels (Lowe et al., 2017). Labels for each pixel sample were determined by combining visual inspection of leaf color with spectral changes (Sun et al., 2018). Specifically, the healthy ROI was defined as the same uninoculated area without any change in the spectrum after inoculation (Figure 3A). The asymptomatic ROI was defined as the area where obvious disease lesions had not yet appeared but the spectral of infected leaves changed. In the asymptomatic stage, light yellowish-green watery lesions could be observed on a few infected leaves, but they are often not easily noticed under field conditions (Figure 3B). The symptomatic ROI was defined as the leaves are turning into wavy yellow green or gray green spots along one or both sides of the leaf margin or along the mid-vein, along with irregular chlorotic spots, and then turning into withered yellow patches or large patches (Figures 3C–E). The boundary between the symptomatic part and the asymptomatic part is observable, whereas it is difficult to distinguish between the asymptomatic and healthy pixels.

### Data Collection and Processing

The configuration of the hyperspectral imaging system and parameter settings in this study can be referred to Zhang et al. (2015). The correlative settings of HSI, including the speed of the motor and positions, could be set by the software (Isuzu Optics Corp, Taiwan, China). The main components and parameters of the hyperspectral imaging system are shown in Table 1.

**TABLE 1:** Main components of the imaging system and parameter settings.

Parameters	Value (unit)	Parameters	Value (unit)
Spectral camera	Raptor EM285	Light input	21V/200W halogen light
Dispersion	97.5 nm/mm	Spectral range	378.28–1033.05 nm
Spectral resolution	2.14 nm	Image size	1632 × 1401 pixels
Spatial resolution	Spot diameter < 9 μm	Object distance	27 cm
Aberration	Halo < 1.5 μm, trapezoid < 1 μm	Exposure time	8 ms
Aperture	F/2.4	Move speed	0.8 mm/s

**FIGURE 4:**
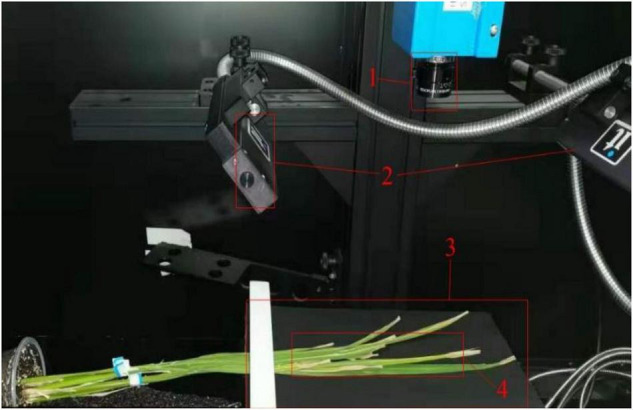
The schematic of the hyperspectral imaging system. 1. Imaging lens; 2. halogen lamps; 3. transportation platform; 4. leaves sample.

The imaging system was pre-heated for 5 min before collecting hyperspectral images. For data collection, rice leaves were placed on the stage below the imaging lens at an object distance of 27 cm, and leaves were fixed on a black background (Figure 4).

The spectrum was recorded in the wavelength range of 378.28–1033.05 nm by the hyperspectral imaging system. In the first conversion step, the information was transformed to a cube format containing the spatial information in the x–y directions and the spectral information in the z direction (Bauriegel et al., 2011). Due to the existence of dark current in the CCD camera and the uneven intensity distribution of the light source in different spectra, the obtained hyperspectral image is unstable. Therefore, the hyperspectral image correction was carried out by the black and white correction method (Zhang et al., 2015). After collecting the hyperspectral images of all diseased rice leaves, the leaf samples were quickly put back into the original environment for further cultivation so as to facilitate the next image collection while reducing the impact of the environment on plant growth and disease development.

### Overview of Data Processing and Modeling Pipeline

**FIGURE 5:**
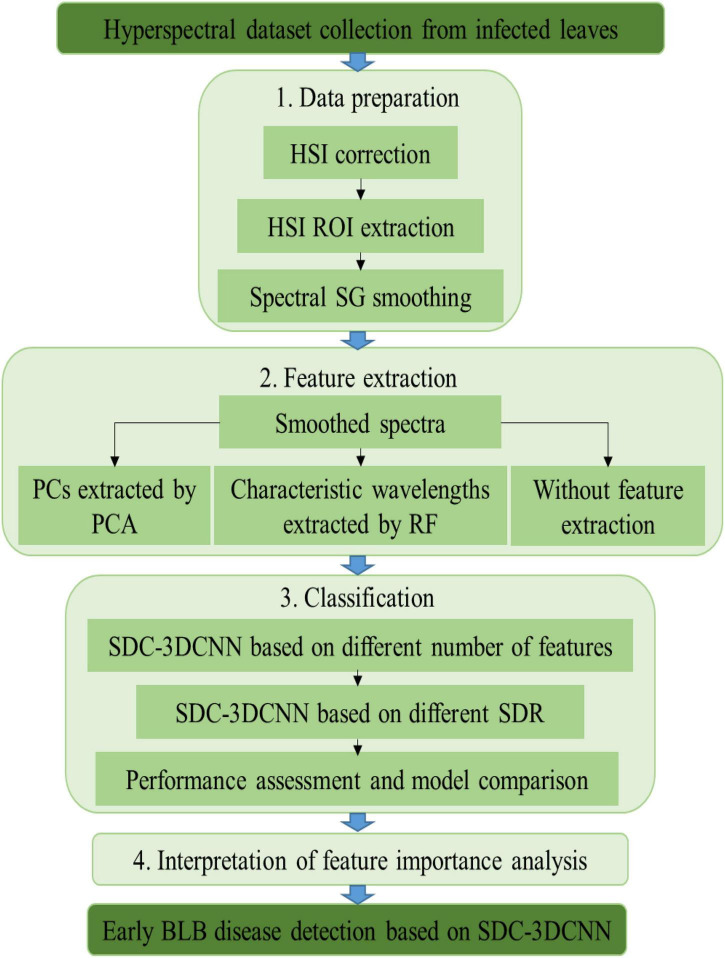
The overview workflow of data processing and modeling pipeline for early rice BLB disease detection.

The spectrum used in this study ranged from 378.28 to 1033.05 nm, 306 wavelengths in total. In order to reduce redundancy and increase the computational efficiency, RF and PCA were used to extract spectral features from raw hyperspectral images. According to the number of extracted features, nine datasets were established. The detection performance of the SDC-3DCNN model over the nine datasets was tested. Meanwhile, the effect of dilation ratios was also examined through experiments. Lastly, the saliency score of wavelength channels was calculated and sorted for interpretation of feature importance. The overview workflow of the data processing and modeling pipeline for early rice BLB disease detection is shown in Figure 5.

#### Hyperspectral Preprocessing

**FIGURE 6:**
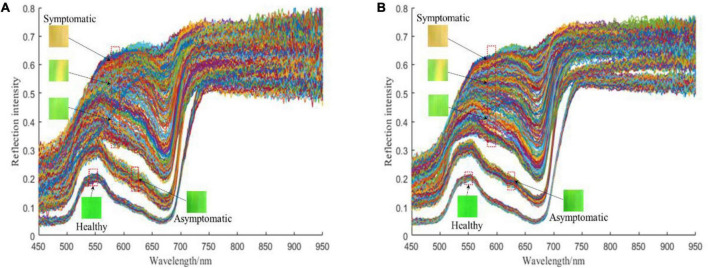
The reflection intensity of different ROIs: **(A)** Original spectra; **(B)** SG smoothed spectra. A single line represents the reflection intensity of an individual spectrum. The total number of spectra in **(A)** and **(B)** is both 1000.

Raw hyperspectral images were analyzed with the software ENVI 5.3 (ITT Visual Information Solutions, Boulder, United States). With this software, symptomatic and healthy pixels of leaves could easily be labeled in a false color image at 450, 550, and 650 nm by visual inspection. While asymptomatic pixels have to be labeled according to the spectral change. The false color images also facilitated the proper manual setting of ROIs and the selection of tissues for spectral analysis. In all hyperspectral images, spectra of healthy and infected tissue areas were obtained pixel-wise. To reduce the impact of noise at both ends of the spectrum, only 450–950 nm (a total of 232 wavelengths) were intercepted for further analysis. The original spectra of different ROIs are shown in Figure 6A.

In order to eliminate the random noise in the spectral signal and improve the signal-to-noise ratio of the sample signal, the Savitzky–Golay (SG) smoothing filter was used to reduce the random noise (the kernel size was 5 × 5 × 5, the polynomial order was 3, and the filter value was calculated at the central node of the kernel). The noise in the spectra of different ROIs was greatly reduced after SG smoothing, and the interference of size difference and different information structures was removed (Figure 6B).

The original spectra and SG smoothed spectra in the range of 450–950 nm are shown in Figure 6. The general distribution of the reflection intensity of the healthy ROI is consistent with the asymptomatic ROI, however, both of which are different from the reflection intensity of the symptomatic ROI. From 450 to 550 nm, the reflectance of all spectra shows an increasing trend. While the trend changes between 550 and 750 nm, for healthy and asymptomatic ROI, the reflectance shows a significant decrease in the spectral range of 550–680 nm and a rapid increase in the range of 680–750 nm. The reflectance of the asymptomatic spectrum is higher at each wavelength than the reflectance of the healthy spectrum. For symptomatic ROI, the reflectance from 680 to 750 nm increases less than the reflectance of asymptomatic ROI. Finally, between 750 and 950 nm, the spectrum tends to be flat. The difference between different spectra provides a basis for classifying different pixels based on the spectral information.

#### Features Extraction by Different Algorithms

Considering that the variation of disease rice physiological parameters could induce strong responses at specific spectral wavelengths, the unbalance of the spectral features selected from the 450–950 nm range could be attributed to the sensitivity of different physiological parameters to disease infection. Spectral information covers wavelengths from 450 to 950 nm and is characterized by a high dimension of redundancy between adjacent wavelengths. Excessive redundant spectral information brings great challenges to detection methods and computational complexity (Hennessy et al., 2020; Zhang et al., 2021b). Therefore, it is necessary to compress the amount of data by a dimensionality reduction method to reduce the cost of subsequent processing on the basis of not dropping the effective feature spectral information (Poona et al., 2016; Sadeghi-Tehran et al., 2021).

**FIGURE 7:**
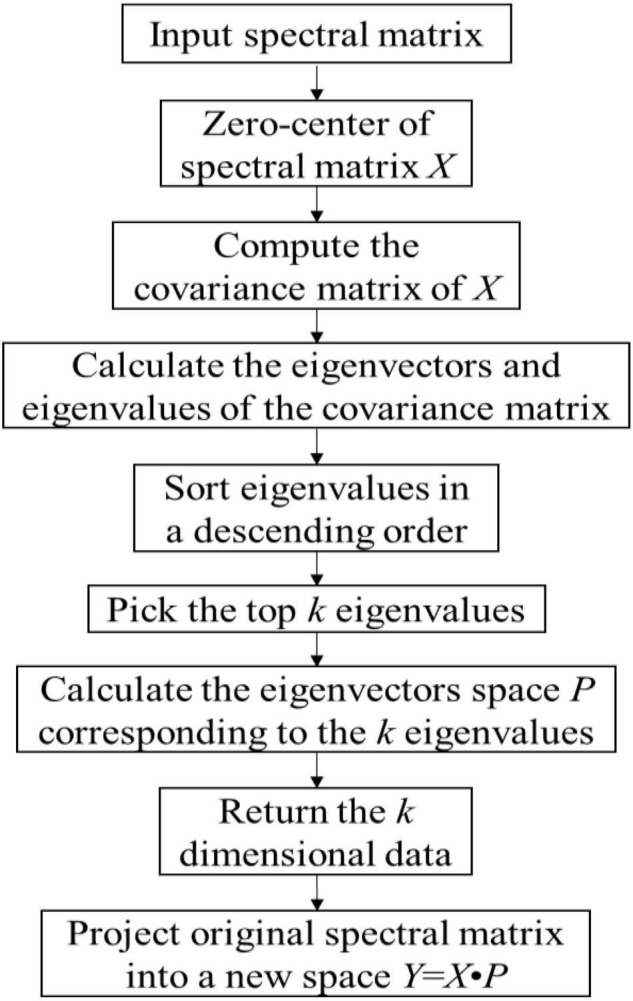
Flowchart of extracting the PCs.

On the one hand, the principal component analysis (PCA) algorithm is a common data compression method and it is often used for dimensionality reduction of high-dimensional data. PCA can extract principal components (PCs) of original hyperspectral data (Hsieh and Kiang, 2020). The main idea of PCA in this paper is to map 232 wavelengths to a k-dimensional feature space (k < 232). On the contrary to simply removing less important dimensional features from the original space, these k-dimensional features can be viewed as PCs through mapping the features in the original space into a latent space. The PCs were obtained by following the calculation process shown in Figure 7.

The PCA was used to extract PCs from the smoothed 232 wavelengths of hyperspectral images. The original hyperspectral data was projected to a k-dimensional space for dimensionality reduction.

On the other hand, the random forest wavelength extraction method is to randomly replace each wavelength and to evaluate its importance (Ma et al., 2018). The higher the wavelength importance, the greater the variation in the prediction error rate would achieve (Hidayat et al., 2017; Speiser et al., 2019). In this way, each wavelength was scored by the change in the error rate of the out-of-bag data before and after wavelength replacement, so as to obtain the characteristic wavelength with a high importance score (Tan et al., 2020). In this paper, in order to reduce the dimension of original hyperspectral data, a different number of characteristic wavelengths were selected according to the important score.

The random forest algorithm used for extracting characteristic wavelengths is described as follows. The number of decision trees in the RF is denoted by *N*_tree_, set at 200; the number of the original wavelength is denoted by *d*, set at 232. The wavelength importance measurement based on out-of-bag error analysis of a single feature *X^j^*(*j* = 1, 2, *d*) was calculated as follows:

Step1: Calculate the number of Out-of-Bag error samples *ErrOOB*_*i*_ of Out-of-Bag data *OOB*_*i*_ corresponding to the *i*^th^ decision tree.

Step2: Keep other wavelengths unchanged, randomly change the wavelength *X^j^* in *OOB*_*i*_, and obtain $OOB_{i}^{j}$.

Step3: Re-calculate the number of out-of-bag error samples $ErrOOB_{i}^{j}$ of out-of-bag data $OOB_{i}^{j}$.

Step4: Repeat steps 1, 2, and 3 to obtain $\left\{ErrOOB_{i}^{j}|i=1,2,,N_{\text{tree}}\right\}$.

Step5: The importance score *VI*(*X^j^*) of wavelength *X^j^* was calculated by Equation 1:


(1) $$VI\left(X^{j}\right)=\frac{1}{N_{tree}}\sum_{i}^{N_{\text{tree}}}\left(ErrOOB_{i}^{j}-ErrOOB_{i}\right)$$

Both PCA and RF were adopted to reduce the dimension of raw hyperspectral data, thereby minimizing the computational complexity. The importance score of each wavelength from the original spectrum was ranked by the above methods, and the top 50, 100, 150, and 200 wavelengths extracted by RF were used to establish four datasets. On the other hand, the principal components (PCs) of the top 50, 100, 150, and 200 rankings extracted from the original spectrum by PCA were used to establish another four datasets. In addition, the original smoothed 232 wavelengths without feature extraction were used to establish the ninth dataset for comparison as well. In total, nine datasets were established.

#### Spectral Dilated Convolution

**FIGURE 8:**
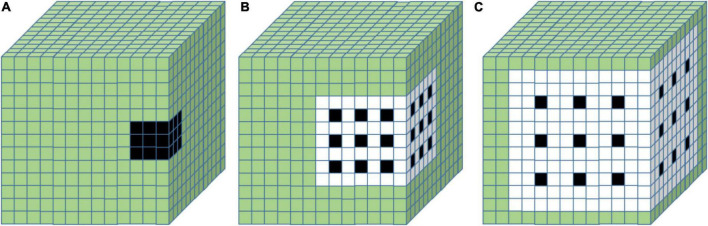
3D dilated convolution under different SDRs: **(A)** SDR = 1; **(B)** SDR = 2; **(C)** SDR = 3. It can be considered that zeros are inserted into the kernel to evenly space the filter under different SDRs.

Dilated convolution is usually applied to expand the receptive field without changing the original structure or the number of parameters of the model (Cao and Guo, 2020). Whereas traditional 2D dilated convolutions can only enlarge the receptive field along spatial dimensions (Xu et al., 2021). Due to the 3D character of hyperspectral data, 3-Dimensional Spectral Dilated Convolution (3D SDC) was developed to expand the receptive field along three dimensions of hyperspectral (Figure 8).

The receptive field of 3D SDC is decided by the spectral dilated ratio (SDR) as:


(2) $$R_{f}=2\times \left(r_{SDR}-1\right)\times \left(k-1\right)+k$$

Where *R_f_* represents the receptive field of a single convolution kernel; *r*_SDR_ represents the spectral dilation ratio; *k* represents the size of convolution kernels. Here *k* was set to 3 (Mohanty et al., 2016; Albattah et al., 2022).

As shown in Figure 8, the black cubes represent the convolution kernel, and the white cubes cover the receptive field. When SDR is set to 1, 2, and 3, the receptive fields of the convolution kernels are 3 × 3 × 3, 7 × 7 × 7, and 11 × 11 × 11, respectively.

#### Residual Module

The increase in the model depth usually improves the performance of the neural network (Xu et al., 2021). However, such an increase may cause gradient vanishing or gradient explosion (Zhong et al., 2018). Cao and Guo (2020) suggested that the 3D residual connection can solve this problem.

The input information can be directly passed to subsequent layers through the residual module. The shortcut connections can be seen as identity mapping. In a residual block, the output of the *l*^th^ block is computed by Equation 3:


(3) $$x_{l+1}=F\left(x_{l}\right)+h\left(x_{l}\right)$$

Where *x_l_* and *x*_*l*1_ are the input and output of the *l*^th^ block, respectively. *F*(*x*_*l*_) is a residual mapping function, and *h*(*x*_*l*_) is an identity mapping function.

**FIGURE 9:**
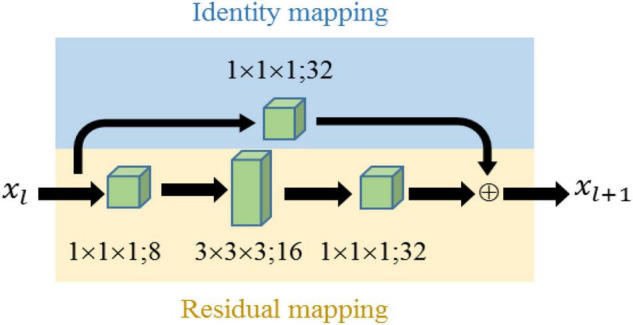
Schematic of the 3D residual module.

As shown in Figure 9, the shortcut connection in the *l*^th^ residual block, shown in blue background, *h*(*x*_*l*_) is basically a direct connection between the input and output of the *l*^th^ block, while *F*(*x*_*l*_) usually contains multiple convolution layers and batch normalization and activation.

#### Spectral Dilated Convolution 3-Dimensional Convolutional Neural Network Framework for Early Disease Detection

**FIGURE 10:**
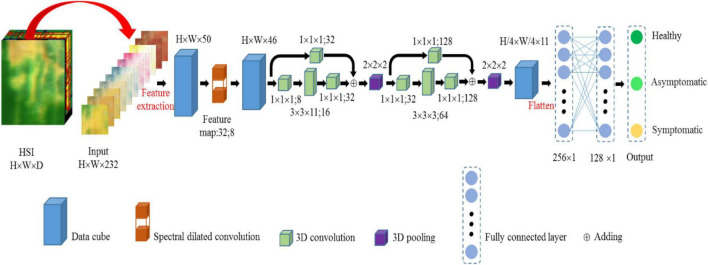
Framework of the spectral dilated convolution 3-dimensional convolutional neural network model.

In this paper, we compared different numbers of input features. The RF can sort the original wavelength according to the importance score. The top 50, 100, 150, and 200 characteristic wavelengths extracted by the RF were treated as inputs to the SDC-3DCNN model, respectively. On the other hand, the top 50, 100, 150, and 200 PCs extracted by PCA from the original spectrum were also treated as inputs to the SDC-3DCNN model, respectively. In addition, the smoothed 232 wavelengths were treated as the input to the SDC-3DCNN model for comparison. The framework of the SDC-3DCNN model is presented in Figure 10, where H, W, and D represent the height, width, and size along the spectral dimension of the data cube. The SDC modules can extract and fuse features corresponding to multiple wavelength resolutions, so the important wavelength information can be more effectively used. We also employed residual blocks to avoid the gradient vanishing problem (Xu et al., 2021). The target sample from the infected rice leaf HSI is composed of 31680 (132 × 240) pixels, which are divided into three classes: healthy, asymptomatic, and symptomatic. The datasets were divided into a training set, a verification set, and a test set under the ratio of 8:1:1.

The 3D convolution is achieved by using a 3D kernel to stack multiple contiguous wavelengths into a cube (Pi et al., 2021). By this construction, the feature maps in the convolution layer are connected to multiple wavelengths frames in the previous layer, thereby capturing spectral information. Formally, the value at position (x, y, z) on the *j*^th^ feature map in the *i*^th^ layer, denoted as $v_{\text{ij}}^{\text{xyz}}$, is computed by Equation 4.


(4) $$v_{ij}^{xyz}=f\left(\sum_{\text{m}}\sum_{h=0}^{h_{i}-1}\sum_{w=0}^{w_{i}-1}\sum_{d=0}^{d_{i}-1}w_{ijm}^{hwd}v_{\left(i-1\right)}^{\left(x+h\right)}m^{\left(y+w\right)\left(Z+d\right)}+b_{ij}\right)$$

Where *H_i_*, *W_i_* and *D_i_* are the height, width, and the size along the spectral dimension of the kernel. $w_{\text{ijm}}^{\text{xyz}}$ is the value at the position (*h*, *w*, *d*) of the kernel connect to the *m*^th^ feature map in the previous layer. *b*_ij_ is the bias for this feature map. *m* indexes over the set of feature maps in the (*i*−1)^th^ layer connected to the current feature map, and the other parameters are the same as in the 2D convolution (Ji et al., 2013).

The SDC-3DCNN model used cross entropy as the loss function and the stochastic gradient descent optimizer for training. The specific parameters of SDC-3DCNN were as follows: learning rate was set to 1 × 10^–3^, weight decay coefficient was set to 1 × 10^–6^, momentum was set to 0.95, epsilon was set to 1 × 10^–5^, epoch was set to 50, dropout was set to 0.45. In order to achieve fast convergence, the training set was divided into multiple batches, and the batch size was set to 64.

### Saliency Wavelengths in Hyperspectral Images

In order to prove the reliability of the detection results, we added post-hoc explanations. A saliency map method was applied to look into the classification results and further improve the overall design of the system. Saliency scores have been used as a popular visualization technique to detect how and why a deep learning neural network makes certain predictions (Zhang et al., 2021c).

In this research, a saliency explanation of wavelength channels was applied. Through one single back-propagation, the derivative ω from a specific predicted result to the input wavelength can be obtained from the well-trained model with the index *h*(*i*, *j*, *c*). Here, (*i*, *j*) indicates the spatial arrangement of elements in ω, while *c* indicates spectral channels (Zhang et al., 2021c). The wavelength saliency scores *W_c_* can be calculated by Equation 5.


(5) $$W_{c}=\sum_{\text{i}}\sum_{\text{j}}\left|\omega_{h\left(i,j,c\right)}\right|$$

All contributions from one specific wavelength channel were summed. The saliency score represents the contribution of different wavelength channels to the classification result. The importance of a specific wavelength can be quantified by the saliency gradient magnitude at that wavelength (Nagasubramanian et al., 2018).

### Classification Assessment

This paper considers the widely acknowledged criteria to evaluate the proposed SDC-3DCNN model, including precision, recall, F1 score, accuracy, and kappa coefficient. True positive, false positive, false negative, and true negative are denoted by TP, FP, FN, and TN, respectively. The formulas of precision (P) and recall (R) are presented in Equations 6 and 7.


(6) $$\text{P}=\frac{\text{TP}}{\text{TP}+\text{FP}}\times 100\%$$

(7) $$\text{R}=\frac{\text{TP}}{\text{TP}+\text{FN}}\times 100\%$$

It is expected that the values of precision and recall should be higher, but they are incompatible. The F1 score is a better metric that combines the characteristics of precision and recall to evaluate the model for different classes in the dataset. A high F1 score is also indicative of satisfactory classification performance. The F1 score formula is presented in Equation 8.


(8) $$\mathrm{F1}=\frac{2\times \text{TP}}{2\times \text{TP}+\text{FN}+\text{FP}}\times 100\%\;$$

Accuracy (A) is another evaluation metric. In general, the higher the accuracy, the better effect that a model would achieve. The formula of accuracy is presented in Equation 9.


(9) $$\text{A}=\frac{\text{TP}+\text{TN}}{\text{TP}+\text{TN}+\text{FN}+\text{FP}}\times 100\%$$

Kappa coefficient (K) is used for consistency test and classification accuracy. The higher the Kappa coefficient, the better consistency that a model would achieve. The formula of Kappa coefficient is presented in Equations 10 and 11:


(10) $$\text{K}=\frac{A-{\text{P}}_{e}}{1-{\text{P}}_{e}}\times 100\%$$

(11) $${\text{P}}_{e}=\frac{\sum_{i=1}^{n}a_{is}\times a_{si}}{{\text{N}}^{2}}$$

Where *a*_is_ is the sum of the elements in the *i* row of confusion matrix, *a*_si_ is the sum of the elements in the *i* column of confusion matrix, *n* is the number of columns in confusion matrix, *N* is the total number of pixel samples.

Besides, the training time and the number of trainable parameters of the model are also important indicators to evaluate the complexity of the model.

## Results

The proposed model is programmed in Python and implemented based on the Tensorflow and Keras open-source deep learning framework. The operating platform hardware configuration includes the NVIDIA GeForce RTX 2080Ti GPU and the AMD Ryzen 5-1600 Six-Core processor @ 3.20 GHZ CPUs.

A total of nine datasets were constructed from 50, 100, 150, and 200 characteristic wavelengths extracted by RF (4 datasets), 50, 100, 150, and 200 PCs extracted by PCA (4 datasets), and smoothed 232 wavelengths without any extraction (1 dataset). Each dataset is divided by 80% for training, 10% for validation, and 10% for testing. The performance of the SDC-3DCNN was evaluated as follows. First, the SDC-3DCNN model was trained and evaluated with those nine datasets. It is noted that at this stage, the SDR was set to 1, meaning that the SDC module did not have any effect. Second, we further explored the effect of the SDC module by adjusting the spectral dilated ratios. Finally, the saliency map method was used to interpret the important features that contributed the most to the classification results.

### Features Extracted by Random Forest and Principal Component Analysis

**FIGURE 11:**
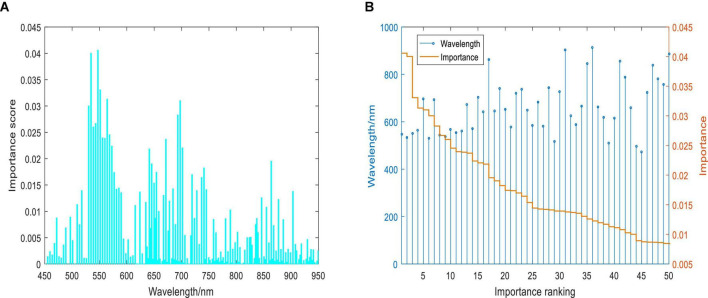
The importance scores of wavelengths: **(A)** importance scores of 232 wavelengths; **(B)** the ranking of importance scores of the top 50 wavelengths.

In this paper, the RF was used to rank the importance of the original 232 wavelengths of the hyperspectral spectrum (Figure 11A). It can be seen that wavelengths with the highest importance scores are mainly distributed at 530–710 nm. It has been shown clearly in Figure 11B that the top 10 wavelengths with high importance scores are 547.2, 534.5, 551.4, 566.2, 697.4, 530.3, 693.0, 543.0, 538.7, and 568.4. The original 232 wavelengths were sorted according to the importance score obtained from the RF. The top 50, 100, 150, and 200 characteristic wavelengths were used to construct datasets for the detection of the asymptomatic BLB disease.

**FIGURE 12:**
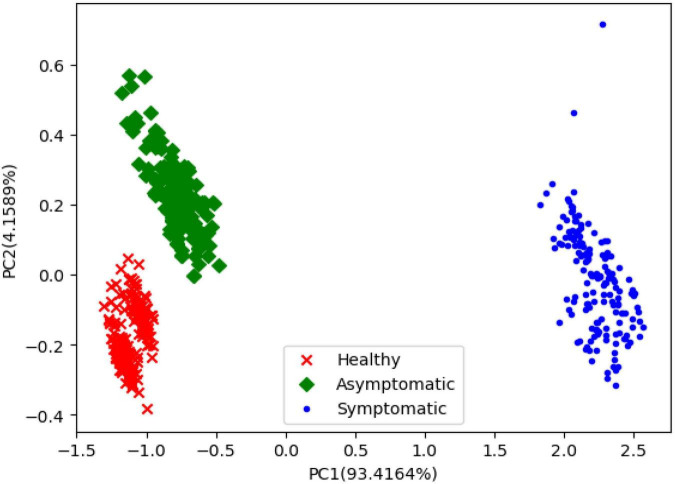
PCA scores map of first two principal components.

On the other hand, the PCA was used to compute the variance contribution of PCs. It can be seen from Figure 12 that the x-axis represents the first principal component score and the y-axis represents the second principal component score. It can be seen that healthy, asymptomatic, and symptomatic pixels were projected into different categories. The top 50, 100, 150, and 200 PCs extracted by PCA were used to establish the datasets, respectively.

### Detection Performance of Spectral Dilated Convolution 3-Dimensional Convolutional Neural Network With Different Input Features

Table 2 shows the performance comparison of using nine datasets to detect healthy, asymptomatic, and symptomatic pixels. The original 232 wavelengths without feature extraction, the top 50, 100, 150, and 200 PCs extracted by PCA, and the top 50, 100, 150, and 200 characteristic wavelengths extracted by RF were used as inputs to the SDC-3DCNN model, respectively. It can be seen from Table 2 that the fewer the input features, the shorter the training time and the less the trainable parameters will be. For the same number of features extracted by RF and PCA, there was a minor difference between the training times. When the number of input features was reduced to 50, the training time is almost reduced to a quarter, and the number of trainable parameters is reduced to about 1/6, compared with the full-feature input. In terms of classification accuracy, when the characteristic wavelengths extracted by RF were used as the input, the overall performance is higher than that when the PCs extracted by PCA and the original 232 wavelengths without features extraction are used as the input. When the input is 50 characteristic wavelengths extracted by RF, the performance of the model achieved the best, with an accuracy of 94.7640% and a kappa coefficient of 92.1466%.

**TABLE 2:** Classification performance of different inputs.

Feature extraction method	Number of input features	Training time/s	Number of trainable parameters	Accuracy/%	Kappa coefficient/%
RF	**50**	**154.1598**	**1,559,731**	**94.7640**	92.1466
	100	263.6340	3,263,667	94.6772	92.0167
	150	382.1071	4,836,531	94.3261	91.4893
	200	502.7476	6,540,467	94.7122	91.0660
PCA	50	154.7476	1,466,515	92.9688	89.4517
	100	262.8174	2,870,451	92.6847	89.0279
	150	383.3145	4,443,315	92.6491	88.9721
	200	501.7399	6,147,251	92.9648	89.4443
None	232	573.6130	7,589,043	89.4492	84.1938

*The bold values denote the achieved best performance.*

**FIGURE 13:**
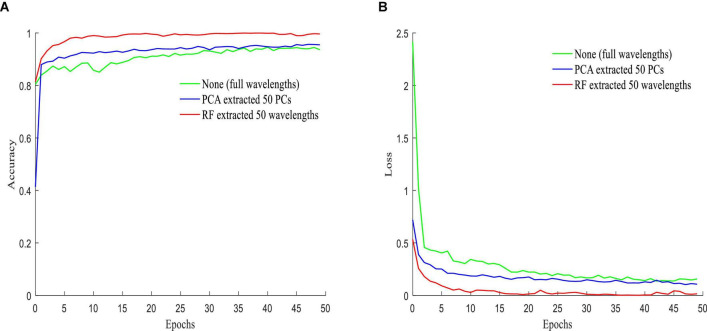
Performance over the validation set: **(A)** accuracy; **(B)** loss.

Figure 13 shows the accuracy (Figure 13A) and loss value (Figure 13B) of the validation set when inputting different features extracted by PCA and RF, as well as the full wavelengths. It can be seen that the worst accuracy was achieved when the full wavelengths were used as the input to the SDC-3DCNN model. However, using features extracted by PCA and RF as inputs, the SDC-3DCNN model achieved better results, especially the latter one.

From the results in Table 2 and Figure 13, it can be seen that the performance is better when the characteristic wavelengths extracted by RF are treated as the input. This shows that the redundant spectral information not only increases the computational complexity and training time of the model but also reduces the performance of the detection model. It can be concluded that RF is more suitable for dimensionality reduction.

### Classification Performance Assessment With Different Spectral Dilated Ratios

It can be seen from Table 2 that when the number of extracted features is 50, the model performance is the best. As a result, we set the number of input features to 50 and then tested the SDR module with various spectral dilated ratios. As shown in Table 3, for the 50 characteristic wavelengths extracted by RF, when the SDR is 5, the SDC-3DCNN model reached the optimal detection performance with an accuracy of 95.4427%. It is also worth noting that when the SDR is set to 5, the SDC-3DCNN model could detect healthy samples more accurately, with a precision of 97.7513%. The performance of detecting symptomatic samples ranks the second, with a precision of 94.3126%. It is nature that the SDC-3DCNN model achieved the worst precision (94.2804%) in the asymptomatic detection task because the asymptomatic samples just had minor differences with the healthy ones. Lastly, it is also glad to notice that the detection performance is generally improved when the SDR is enabled (when SDR is set greater than 1).

**TABLE 3:** Performance of spectral dilated convolution 3-dimensional convolutional neural network models under different SDRs with RF extracted feature inputs.

Class	Evaluating indicator	SDR				
		1	2	3	4	5
Healthy	Precision/%	94.7901	98.8107	97.3088	97.3327	97.7513
	Recall/%	99.4036	96.1350	98.3419	98.3777	98.0079
	F1-score/%	97.0420	97.4545	97.8226	97.8524	97.8794
Asymptomatic	Precision/%	92.5733	90.5362	96.8478	91.1809	94.2804
	Recall/%	91.4542	95.3800	86.4153	94.5302	91.7415
	F1-score/%	92.0104	92.8950	91.3346	92.7431	92.9936
Symptomatic	Precision/%	96.9153	96.5538	90.3685	97.1809	94.3126
	Recall/%	93.4581	94.0855	98.8845	92.5285	96.5373
	F1-score/%	95.1553	95.3037	94.4349	94.7976	95.4120
Average precision/%		94.7596	95.3002	94.8417	95.2315	**95.4481**
Average recall/%		94.7720	95.2002	94.5472	95.1455	**95.4289**
Average F1-score/%		94.7359	95.2177	94.5307	95.1310	**95.4283**
Accuracy/%		94.7640	95.1902	94.5944	95.1231	**95.4427**

*The bold values denote the achieved best performance.*

It can be seen from Table 4, for 50 PCs extracted by PCA as the input, when the SDR is 3, the detection performance reached the best, and the accuracy achieved at 93.2252%. Diving into the detection performance of each class, it is noted that the SDC-3DCNN model still can predict the healthy samples with the highest precision (96.6517%), following the symptomatic class with a precision of 93.2132%. This finding indicated that the class sensitivity varies in the SDR settings. Meanwhile, we noticed that when the 50 PCs were treated as the input and the SDR was set to 2, the precision of detecting asymptomatic samples reached at 91.1620%, even higher than the performance of symptomatic detection.

**TABLE 4:** Performance of spectral dilated convolution 3-dimensional convolutional neural network models under different SDRs with PCA extracted feature inputs.

Class	Evaluating indicator	SDR				
		1	2	3	4	5
Healthy	Precision/%	96.4727	96.8532	96.6517	96.9132	96.5840
	Recall/%	97.8767	97.6619	97.4472	97.3756	97.1371
	F1-score/%	97.1696	97.2559	97.0478	97.1439	96.8598
Asymptomatic	Precision/%	90.2768	91.1620	89.7676	88.7874	89.8821
	Recall/%	88.2346	86.6667	89.6709	89.5631	87.6122
	F1-score/%	89.2440	88.8575	89.7192	89.1736	88.7327
Symptomatic	Precision/%	92.0673	90.5007	93.2132	92.7705	91.4471
	Recall/%	92.7841	94.0971	92.5633	91.5524	93.1792
	F1-score/%	92.4243	92.2639	92.8871	92.1574	92.3050
Average precision/%		92.9389	92.8386	**93.2108**	92.8237	92.6377
Average recall/%		92.9651	92.8086	**93.2271**	92.8304	92.6428
Average F1-score/%		92.9460	92.7924	**93.2180**	92.8250	92.6325
Accuracy/%		92.9688	92.8267	**93.2252**	92.8227	92.6531

*The bold values denote the achieved best performance.*

By summarizing the results in Tables 3, 4 it can be concluded that compared with using the 50 PCs extracted by PCA as the input, using the 50 wavelengths extracted by RF may generally achieve better performance, no matter how the SDR was set. Meanwhile, due to the feature information extracted by RF and PCA is different, the SDC-3DCNN model requires specific SDR settings.

**FIGURE 14:**
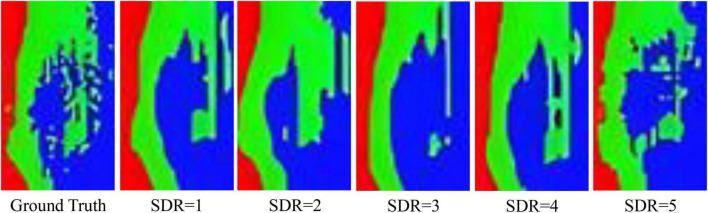
Comparative results under different SDRs. The red, green, and blue pixels represent healthy, asymptomatic, and symptomatic pixels, respectively. The input to the spectral dilated convolution 3-dimensional convolutional neural network model is 50 characteristic wavelengths extracted by RF.

For further exploring the effect of SDR settings, we presented the detection results in Figure 14. The red, green, and blue pixels represent healthy, asymptomatic, and symptomatic pixels, respectively. It can be seen that when the SDR equals 1–4, the SDC-3DCNN model failed to classify certain pixels, especially the ones near the boundary between asymptomatic and symptomatic areas. This can be attributed to the over-extraction of the spectral information. When SDR equals 5, the classification result was fairly good, as more pixels can be classified correctly.

### Interpretation of Feature Importance Analysis

**FIGURE 15:**
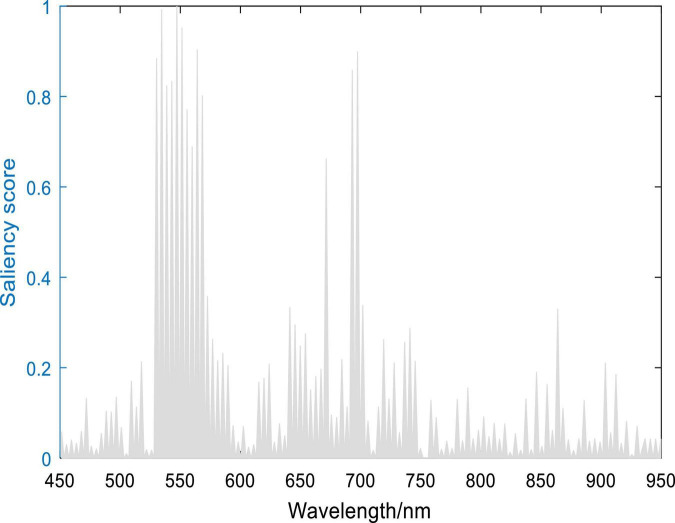
Spectral saliency scores.

Figure 15 shows the saliency scores of each wavelength channel, which guides us to extract the important channels for detecting healthy, asymptomatic, and symptomatic pixels from infected rice leaves.

**FIGURE 16:**
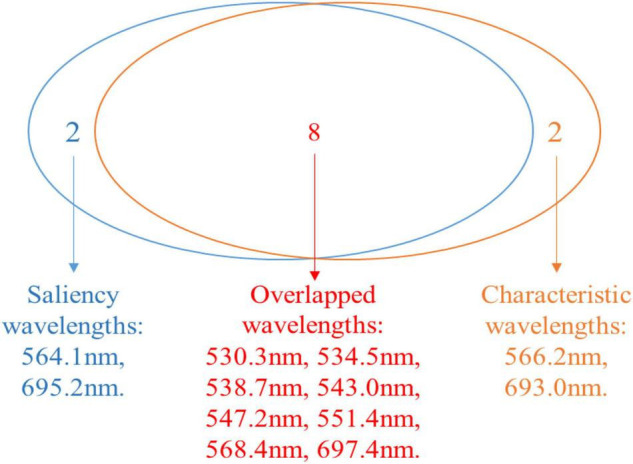
Venn diagram for the characteristic wavelengths and the saliency wavelength channels.

The contributions of different wavelengths are depicted as the gray area shown in Figure 15. Here the spectral saliency scores were min-max normalized into the range of [0, 1]. It can be seen that all channels did not contribute equally to the classification result. The regions between 530–580 and 680–710 nm contributed the most, which were 547.2, 534.5, 551.4, 564.1, 697.4, 530.3, 695.2, 543.0, 538.7, and 568.4 nm. As shown in Figure 16, when compared with the top 10 characteristic wavelengths extracted by RF, it can be found that eight characteristic wavelengths overlapped with the saliency sensitive wavelengths. Through analysis of interpretation, the accuracy of the extracted wavelengths by RF is verified. At the same time, the saliency score can also be used to determine the characteristic wavelengths and reduce the dimensionality of the spectrum, thereby improving the computational efficiency. This evidence also provides some explanations for the decision-making of the model, indicating that the model might use these features to distinguish pixels in different infected states.

## Discussion

### Advantages of Using Feature Extraction and Spectral Dilated Convolution 3-Dimensional Convolutional Neural Network Classification Models

Our results suggest that the combination of deep learning and hyperspectral imaging can be a useful approach for detecting asymptomatic infections of BLB disease in rice leaves. Previous studies have demonstrated that redundancy exists in the raw hyperspectral spectrum, leading to an increase in computational complexity (Li et al., 2017). Therefore, feature extraction is necessary. In this study, RF and PCA both can extract characteristic features from the raw hyperspectral data. Additionally, the characteristic wavelengths extracted by RF may assist the SDC-3DCNN model in achieving better classification performance than the ones extracted by PCA. Compared with PCA, the characteristic wavelengths extracted by RF could be better interpreted. The advantage of interpretability enables a multispectral camera to incorporate only the most important wavelengths instead of adopting the full wavelengths for disease detection.

### Feasibility of Hyperspectral in Asymptomatic Disease Detection

We find that the healthy, asymptomatic, and symptomatic pixels of rice leaves are sensitive to different wavelengths. According to Šebela et al. (2018), Zhang S. et al. (2019), the invasion of the rice BLB disease will cause inner changes such as pigment, cell structure, and leaf water content. The reflectance of different pixels is mainly affected by those inner changes in leaves, which provides a theoretical basis for disease detection based on hyperspectral imaging. In recent studies (Su et al., 2018; Wang et al., 2019; Savian et al., 2020), it has been demonstrated that accurate extraction of leaf characteristic wavelengths at different scales is feasible and facilitates the possibility of detecting the subtle spectral variation induced by the BLB disease infection. Compared with RGB images, hyperspectral imaging can obtain not only the texture and color information, but also the spectral information of the internal changes in rice leaves. This early detection can guide growers to prevent and apply pesticides in advance, thereby avoiding the BLB disease outbreak. On the other hand, the asymptomatic detection method proposed in this paper for the BLB disease can also be applied to the detection of asymptomatic infection of other plant diseases.

### Potential of Spectral Dilated Convolution for Hyperspectral Imaging

At present, dilated convolution is mostly applied to increase the receptive field without pooling in 2D computer vision tasks. For the same feature map, a larger receptive field can decrease the computation complexity and improve the effect of small object recognition. Whereas the hyperspectral image is a cube, which not only includes 2D spatial information but also includes the spectral information, 3D spectral dilated convolution is needed for hyperspectral image processing. There is rarely research on 3D spectral dilated convolution for hyperspectral data analysis. Xu et al. (2021) used 3D dilated convolution in hyperspectral image classification and achieved a good result. However, only spectral dimension dilated convolution was considered in the research. Considering the 3D characteristics of hyperspectral images and the information redundancy of the spectral dimension, we tested the effectiveness of 3D SDC in hyperspectral data processing. Different SDRs were compared and the SDC-3DCNN model achieved the best performance when the SDR was 5. The 3D SDC increases the receptive field not only in the spectral dimension but also in the spatial dimension, thus obtaining richer features.

### Interpretation of Characteristic Wavelengths

At current, researches are paying attention to the interpretability of deep learning models because the explainable scale network can more precisely analyze the relationship between the learned scale features and different classes (Shi et al., 2022). Human-understandable results are more acceptable than undetectable black-box results, especially in the practical application of disease detection. On the basis of spectral saliency, we can find that the important wavelengths for classifying healthy, asymptomatic, and symptomatic pixels were located at 530-710 nm, which is consistent with the research results of Wang et al. (2019). In this paper, we used the saliency score to interpret the results of the model and infer the wavelengths that contributed the most to the output results. The top 10 saliency wavelengths overlapped with 8 wavelengths from the top 10 characteristic wavelengths extracted by RF, which indicated the validity of RF in extracting characteristic wavelengths. On the other hand, the saliency map method can figure out the important wavelengths, which are not extracted by RF. In addition to the significant saliency score, other interpretation methods can be further developed.

## Conclusion

This is the first study to use hyperspectral imaging and deep learning to detect the infection of rice leaf BLB disease, particularly in the early asymptomatic stage that RGB imaging cannot detect. In this paper, RF and PCA were used to extract features from raw hyperspectral data. The detection performance of the SDC-3DCNN model with different input features and spectral dilated ratios was tested and compared. When 50 wavelengths extracted by RF were used as the input and SDR was set to 5, the SDC-3DCNN model achieved the highest accuracy at 95.4427%. In addition, the effectiveness of extracting characteristic wavelengths was verified by saliency scores, and the wavelengths with the greatest contributions were in the range of 530–710 nm. In conclusion, the combination of deep learning and hyperspectral imaging can achieve good performance for asymptomatic rice BLB disease detection. The proposed method can further evaluate the incidence of plant diseases, providing an early disease warning for farmers to apply pesticides accurately and efficiently.

## Data Availability Statement

The raw data supporting the conclusions of this article will be made available by the authors, without undue reservation.

## Author Contributions

YC contributed to the conceptualization, methodology, software, data curation, writing original draft preparation, reviewing and editing. ZZ and HX contributed to the supervision, project administration, funding acquisition, writing - review and editing. JM-O contributed to the conceptualization and methodology. PY and JF contributed to the investigation, data curation, and resources. All authors read and approved the final manuscript.

## Conflict of Interest

The authors declare that the research was conducted in the absence of any commercial or financial relationships that could be construed as a potential conflict of interest.

## Publisher’s Note

All claims expressed in this article are solely those of the authors and do not necessarily represent those of their affiliated organizations, or those of the publisher, the editors and the reviewers. Any product that may be evaluated in this article, or claim that may be made by its manufacturer, is not guaranteed or endorsed by the publisher.
